# Disrupting neuronal transmission: mechanism of DBS?

**DOI:** 10.3389/fnsys.2014.00033

**Published:** 2014-03-14

**Authors:** Satomi Chiken, Atsushi Nambu

**Affiliations:** Division of System Neurophysiology, National Institute for Physiological Sciences and Department of Physiological Sciences, Graduate University for Advanced StudiesMyodaiji, Okazaki, Japan

**Keywords:** deep brain stimulation, basal ganglia, subthalamic nucleus, globus pallidus, cortico-basal ganglia loop, electrophysiology

## Abstract

Applying high-frequency stimulation (HFS) to deep brain structure, known as deep brain stimulation (DBS), has now been recognized an effective therapeutic option for a wide range of neurological and psychiatric disorders. DBS targeting the basal ganglia thalamo-cortical loop, especially the internal segment of the globus pallidus (GPi), subthalamic nucleus (STN) and thalamus, has been widely employed as a successful surgical therapy for movement disorders, such as Parkinson’s disease, dystonia and tremor. However, the neurophysiological mechanism underling the action of DBS remains unclear and is still under debate: does DBS inhibit or excite local neuronal elements? In this review, we will examine this question and propose the alternative interpretation: DBS dissociates inputs and outputs, resulting in disruption of abnormal signal transmission.

## Introduction

Applying high-frequency electrical stimulation (HFS) to a specific target in subcortical structures, known as deep brain stimulation (DBS), was introduced as a surgical treatment for movement disorders in early 1990s (Benabid et al., 1991, 1994; Siegfried and Lippitz, 1994a,b; Limousin et al., 1995). Since then, DBS has been widely accepted as an effective therapeutic option. DBS targeting the ventral thalamus dramatically alleviates essential and resting tremor (Benabid et al., 1991, 1996; Siegfried and Lippitz, 1994b; Koller et al., 1997; Rehncrona et al., 2003). DBS targeting the subthalamic nucleus (STN) and the internal segment of the globus pallidus (GPi) has been largely used for treatment of Parkinson’s disease, and GPi-DBS has marked effects on improvement of dystonic symptoms (Limousin et al., 1995; Deep-Brain Stimulation for Parkinson’s Disease Study Group, 2001; Coubes et al., 2004; Wichmann and Delong, 2006; Kringelbach et al., 2007; Ostrem and Starr, 2008; Vitek, 2008; Vidailhet et al., 2013). However, the exact mechanism of the effectiveness remains to be elucidated.

Since DBS gives rise to similar effects to those of lesions, it was originally considered to inhibit local neuronal elements. In fact, neuronal firings of neighboring neurons were inhibited by STN- or GPi-DBS (Boraud et al., 1996; Dostrovsky et al., 2000; Wu et al., 2001; Filali et al., 2004; Lafreniere-Roula et al., 2010). On the other hand, recent studies have emphasized activation of neuronal elements. Actually, STN-DBS increased activity of GPi neurons through the excitatory STN-GPi projections (Hashimoto et al., 2003; Galati et al., 2006; Reese et al., 2011), and GPi-DBS reduced activity of thalamic neurons through the inhibitory GPi-thalamic projections (Anderson et al., 2003; Pralong et al., 2003; Montgomery, 2006). In addition, recent studies reported multi-phasic responses consisting of excitation and inhibition in GPi neurons during GPi-DBS (Bar-Gad et al., 2004; Erez et al., 2009; McCairn and Turner, 2009; Leblois et al., 2010). In this article, we critically review recent studies, and discuss the possible mechanism of effectiveness of DBS.

## Deep brain stimulation (DBS) inhibits local neuronal elements

Both DBS and lesion were found to produce similar benefits on alleviation of symptoms. For example, STN-DBS has similar effects on Parkinsonian motor signs (Benazzouz et al., 1993; Benabid et al., 1994; Limousin et al., 1995) to the STN-lesion (Bergman et al., 1990; Aziz et al., 1991; Levy et al., 2001) and blockade of synaptic transmission from the STN to the GPi (Graham et al., 1990; Brotchie et al., 1991). Thus, DBS was originally assumed to inhibit local neuronal elements. Actually, the most common effect of STN- or GPi-HFS on neighboring neurons was reduction of the firing rates.

Distinct suppression of neuronal activity was recorded during STN-DBS around the stimulating sites in Parkinsonian patients during stereotactic surgery (Filali et al., 2004; Welter et al., 2004). Similar results were also obtained in animal models, such as Parkinsonian monkeys (Meissner et al., 2005; Moran et al., 2011) and rats (Tai et al., 2003; Shi et al., 2006). Stimulus artifacts hinder detection of spikes during 2–3 ms after stimulus pulses and some spikes may be obscured when neuronal activities are recorded nearby the stimulating electrodes. Recent studies enabled detection of spikes just after stimulus pulses by removal of stimulus artifacts using the template subtraction method (Wichmann, 2000; Hashimoto et al., 2002) and confirmed that STN-DBS decreased firing of neighboring neurons (Meissner et al., 2005; Moran et al., 2011). Although STN-HFS much decreased neuronal firing around the stimulation site, complete cessation of STN firing was observed in a limited number of neurons. STN-HFS at 140 Hz reduced mean firing rate of STN neurons by 77% in Parkinsonian patients, and among them, 71% of STN neurons exhibited residual neuronal activity, while only 29% of STN neurons exhibited total inhibition (Welter et al., 2004). Similar results were also observed in Parkinsonian monkeys (Meissner et al., 2005), and Parkinsonian and normal rats (Tai et al., 2003). Decreased abnormal oscillatory activity in the STN was also observed during STN-DBS in Parkinsonian monkeys (Meissner et al., 2005). Inhibitory effects sometimes outlasted the stimulus period (Tai et al., 2003; Filali et al., 2004; Welter et al., 2004).

**Figure 1:**
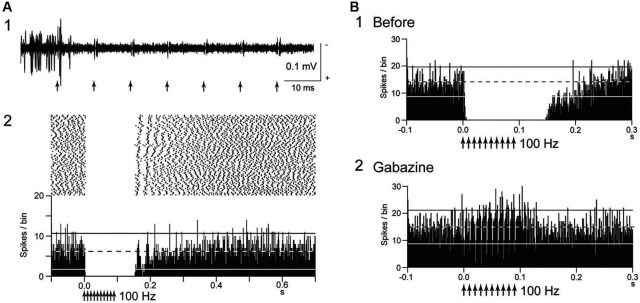
**Deep brain stimulation (DBS) inhibits local neuronal firing. (A)** Responses of an internal pallidal (GPi) neuron to local GPi repetitive high-frequency stimulation (HFS; 30 μA, 100 Hz, 10 pulses) in a normal monkey. Raw traces of spike discharges after removing the stimulus artifacts by the template subtraction method **(1)** and raster and peristimulus time histogram (PSTHs; 100 trials; binwidth, 1 ms) **(2)** are shown. Arrows indicate the timing of local stimulation. Spontaneous discharges of the GPi neuron were completely inhibited by the stimulation. **(B)** Effect of local injection of gabazine (GABA_A_ receptor antagonist) in the vicinity of the recorded GPi neuron on inhibition of spontaneous activity induced by GPi-HFS. The inhibition was abolished after gabazine injection. Modified from Chiken and Nambu (2013).

Inhibitory effects of GPi-DBS on firing of the neighboring neurons were also reported (Boraud et al., 1996; Dostrovsky et al., 2000; Wu et al., 2001; McCairn and Turner, 2009). Complete inhibition of local neuronal firing was more commonly induced by GPi-DBS than by STN-DBS (Figure 1A). GPi-HFS at 100 Hz induced complete inhibition of 76% of neighboring neurons in normal monkeys (Chiken and Nambu, 2013), and the inhibition outlasted the stimulus period, sometimes over 100 ms after the end of stimulation. Similar post-train inhibition was also observed in Parkinsonian patients (Lafreniere-Roula et al., 2010).

To the contrary, multiphasic responses consisting of the excitation and inhibition during GPi-HFS were recently observed in GPi neurons of Parkinsonian monkeys (Bar-Gad et al., 2004; Erez et al., 2009; McCairn and Turner, 2009) and dystonic hamsters (Leblois et al., 2010). The discrepant results may be due to differences in stimulus parameters used in these experiments: larger axons are easily activated by electrical stimulation than smaller ones (Ranck, 1975), and continuous repetitive stimulation might cause failure of postsynaptic events due to receptor desensitization and/or transmitter depletion (Wang and Kaczmarek, 1998; Zucker and Regehr, 2002). Such multiphasic responses may normalize abnormal firings, such as bursting and oscillatory activity in Parkinson’s disease and dystonia as described below.

## Mechanism of inhibition

**Figure 2:**
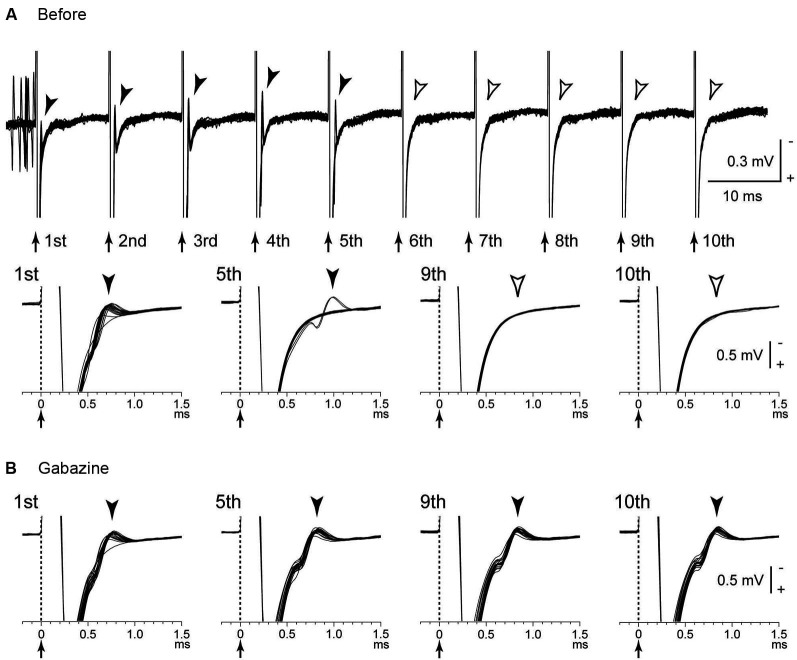
**Directly evoked spikes of GPi neurons were inhibited during GPi-HFS. (A)** Raw traces showing directly evoked spikes of a GPi neuron by stimulus pulses during GPi-HFS (40 μA, 100 Hz, 10 pulses) in a normal monkey. Traces with long (top) and short (bottom) time scales are shown. Arrows with dotted lines indicate the timing of local stimulation (time 0 in the bottom traces). Filled arrowheads indicate directly evoked spikes. GPi-HFS failed to evoke spikes (open arrowheads; from 6th to 10th stimuli). **(B)** Effects of local gabazine injection on the inhibition of direct evoked GPi responses. Gabazine injection decreased failure rate, and each stimulus successfully evoked spikes (5th, 9th, and 10th stimuli). Modified from Chiken and Nambu (2013).

Several possible mechanisms account for the inhibitory responses have been proposed, including depolarization-block and inactivation of voltage-gated currents (Beurrier et al., 2001; Shin et al., 2007). However, these are less probable, because both single-pulse and low-frequency stimulation in the GPi evoked intense short latency inhibition in neighboring neurons (Dostrovsky et al., 2000; Dostrovsky and Lozano, 2002; Chiken and Nambu, 2013). Another possible mechanism is that the inhibition is caused by activation of GABAergic afferents in the stimulated nucleus (Boraud et al., 1996; Dostrovsky et al., 2000; Dostrovsky and Lozano, 2002; Meissner et al., 2005; Johnson et al., 2008; Liu et al., 2008; Deniau et al., 2010). A recent study confirmed that inhibitory responses induced by GPi-HFS were mediated by GABA_A_ and GABA_B_ receptors (Chiken and Nambu, 2013; Figure 1B). GABAergic inhibition is strong and inhibits even directly evoked spikes by GPi stimulation, which is characterized constant- and short latency (Figures 2A, B; Chiken and Nambu, 2013).

The GPi receives excitatory glutamatergic inputs from the STN as well as inhibitory GABAergic inputs from the striatum and GPe (Smith et al., 1994; Shink and Smith, 1995). Afferent axon terminals from the STN are also activated by the stimulation, but the glutamatergic excitation is probably overwhelmed because of predominance of GABAergic inputs in the GPi (Shink and Smith, 1995). On the other hand, many GPe neurons exhibited complex responses composed of both excitation and inhibition during GPe-HFS (Chiken and Nambu, 2013). The density of GPe terminals on GPi neurons is higher than those on GPe neurons (Shink and Smith, 1995), and the balance between GABAergic and glutamatergic inputs may explain the different effects between GPe-HFS and GPi-HFS. Similarly, STN-HFS stimulated both glutamatergic and GABAergic afferents and generated both excitatory and inhibitory post-synaptic potentials (EPSPs and IPSPs) in the STN neurons (Lee et al., 2004). Thus, HFS activates afferent axons in the stimulated nucleus, and the effects vary depending on the composition of the inhibitory and excitatory axon terminals.

## Deep brain stimulation (DBS) excites local neuronal elements

It is rational that local stimulation excites local neuronal elements. Actually, directly evoked spikes, which are characterized by short- and constant latency, are induced in GPi neurons by GPi-HFS (Johnson and McIntyre, 2008; McCairn and Turner, 2009). Such excitation may propagate through efferent projections. Thalamic activity was reduced during GPi-HFS through inhibitory GPi-thalamic projections in Parkinsonian monkeys (Anderson et al., 2003) and dystonia patients (Pralong et al., 2003; Montgomery, 2006). GPi activity was increased during STN-DBS through excitatory STN-GPi projections (Hashimoto et al., 2003; Galati et al., 2006; Reese et al., 2011). STN-DBS increased both glutamate and GABA levels in the substantia nigra pars reticulata (SNr) of normal rats in microdialysis studies (Windels et al., 2000; see also Windels et al., 2005). An intraoperative microdialysis study revealed that STN-DBS produced significant increase in extracellular concentration of cyclic guanosine monophosphate (cGMP) in the GPi (Stefani et al., 2005). Functional magnetic resonance imaging (MRI) and positron emission tomography (PET) studies in humans indicated that efferent outputs from the stimulated nucleus are excited during DBS (Jech et al., 2001; Hershey et al., 2003; Boertien et al., 2011). Changes of the firing rates and patterns of target nuclei may normalize abnormal firings, such as bursting and oscillatory activity, which are observes in the cortico-basal ganglia loop of Parkinson’s disease and dystonia (Anderson et al., 2003; Hashimoto et al., 2003; Hammond et al., 2007; Johnson et al., 2008; Vitek, 2008; Deniau et al., 2010).

According to the modeling study (McIntyre et al., 2004), subthreshold HFS suppressed intrinsic firings in the cell bodies, while suprathreshold HFS generated efferent outputs at the stimulus frequency in the axon without representative activation of the cell bodies. Thus, although stimulation may fail to activate cell bodies of GPi neurons due to strong GABAergic inhibition, it can still excite the efferent axons and provide inhibitory inputs to the thalamus at the stimulus frequency.

DBS also antidromically excites afferent axons. Actually, antidromic activation of GPi neurons induced by STN-DBS was observed in Parkinsonian monkeys (Moran et al., 2011), and antidromic activation of thatamic (Vop) neurons induced by GPi-DBS was observed in Parkinsonian patients (Montgomery, 2006). Low intensity STN-HFS induced GABAergic inhibition in the SNr through antidromic activation of GPe neurons projecting to both the STN and SNr (Maurice et al., 2003; see also Moran et al., 2011), whereas higher intensity stimulation induced glutamatergic excitation in the SNr through activation of STN-SNr projections. STN-HFS also activated motor cortical neurons antidromically and suppressed abnormal low frequency synchronization including beta band oscillation in Parkinsonian rats (Li et al., 2007, 2012; Degos et al., 2013). Recent development of optogenetics has enabled selective stimulation of afferent inputs or efferent outputs, and contribute to analyzing the mechanism of effectiveness of DBS. A recent study has shown that selective stimulation of cortico-STN afferent axons can robustly ameliorate symptoms in Parkinsonian rats without activation of STN efferent axons (Gradinaru et al., 2009), suggesting that therapeutic effects of STN-DBS may be exclusively accounted for activation of cortico-STN afferent axons.

It is also probable that STN-DBS induces dopamine release through STN-SNc projections. STN-DBS induced dopamine release by activation of nigrostriatal dopaminergic neurons in rats (Meissner et al., 2003) and pigs (Shon et al., 2010), however it did not increase dopamine level of the striatum in human patients (Abosch et al., 2003; Hilker et al., 2003). DBS may also affect neurons whose axons pass nearby the stimulating site. A model-based study showed that clinically effective STN-DBS also activated the lenticular fasciculus, which is composed of GPi-thalamic fibers, in addition to STN neurons temselves (Miocinovic et al., 2006). Actually, STN-DBS induced direct excitation of GPi neurons through activation of the lenticular fasciculus (Moran et al., 2011).

Participation of non-neuronal glial tissues should also be considered as one of possible mechanisms of DBS effectiveness. DBS induced glutamate and adenosine triphosphate (ATP) release from astrocytes (Fellin et al., 2006; Tawfik et al., 2010). A recent study revealed that HFS applied to the thalamus induced abrupt increase in extracellular ATP and adenosine (Bekar et al., 2008). Adenosine activation of A1 receptors depressed excitatory transmission in the thalamus, and alleviated tremor in a mouse model. Thus, it is possible that ATP and glutamate are released from astrocytes triggered by DBS and modulate neuronal activity in the stimulated nucleus (Vedam-Mai et al., 2012; Jantz and Watanabe, 2013).

## Deep brain stimulation (DBS) disrupts neuronal transmission

**Figure 3:**
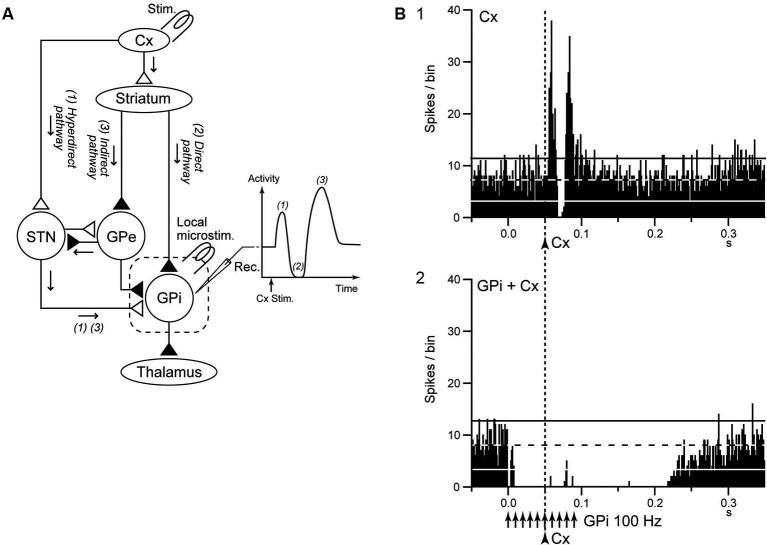
**GPi-DBS disrupts information flow through the GPi. (A)** Schematic diagram showing the cortico-basal ganglia pathway and stimulating (Stim.) and recording (Rec.) sites in the electrophysiological experiments (left), along with a typical response pattern (right) in the (GPi) to cortical stimulation (Cx Stim.) with early excitation, inhibition, and late excitation, which are mediated by the **(1)** cortico-subthalamo (STN)-GPi *hyperdirect*, **(2)** striato-GPi *direct*, and **(3)** striato-external pallido (GPe)-STN-GPi *indirect* pathways, respectively. **(B)** Effects of local GPi-HFS on cortically evoked responses of a GPi neuron in a normal monkey. PSTH (100 trials) in response to the single pulse stimulation (arrowhead with dotted line) of the primary motor cortex (Cx) **(1)** and PSTH in response to Cx stimulation (arrowhead with dotted line) during GPi-HFS (arrows) **(2)** are shown. Cortical stimulation was applied 50 ms after the initiation of GPi-HFS. The cortically evoked responses were entirely inhibited during GPi-HFS. Modified from Chiken and Nambu (2013).

The striatum and STN are input stations of the basal ganglia and receive inputs from a wide area of the cerebral cortex (Mink, 1996; Nambu et al., 2002). The information is processed through the *hyperdirect*, *direct*, and *indirect* pathways and reaches the GPi/SNr, the output station of the basal ganglia (Figure 3A). During voluntary movements, neuronal signals originating in the cortex are considered to be transmitted through these pathways, reach the GPi/SNr and control movements (Mink, 1996; Nambu et al., 2002). Signal transmission through the *direct* pathway reduces GPi activity and facilitates movements by disinhibiting the thalamus, whereas the *hyperdirect* and* indirect* pathways increase GPi activity and suppress movements (Nambu et al., 2002; Nambu, 2007; Kravitz et al., 2010; Sano et al., 2013).

Chiken and Nambu (2013) recently examined responses of GPi neurons evoked by motor cortical stimulation during GPi-HFS in normal monkeys. In that study, both cortically evoked responses and spontaneous discharges were completely inhibited during GPi-HFS by strong GABAergic inhibition (Figure 3B), suggesting that GPi-HFS blocks information flow through the GPi. Since abnormal cortically evoked responses (Chiken et al., 2008; Kita and Kita, 2011; Nishibayashi et al., 2011) and abnormal bursts and oscillatory activity (Wichmann et al., 1994; Bergman et al., 1998; Starr et al., 2005; Brown, 2007; Chiken et al., 2008; Nishibayashi et al., 2011; Tachibana et al., 2011) were observed in GPi neurons in Parkinson’s disease and dystonia, signal transmission of such abnormal activities to the thalamus and motor cortex would be responsible for motor symptoms. Thus, disruption of the abnormal information flow could suppress expression of motor symptoms. This mechanism may explain the paradox that GPi-DBS produces similar therapeutic effects to lesions of the GPi: both GPi-DBS and GPi-lesion interrupt abnormal information flow through the GPi.

**Figure 4:**
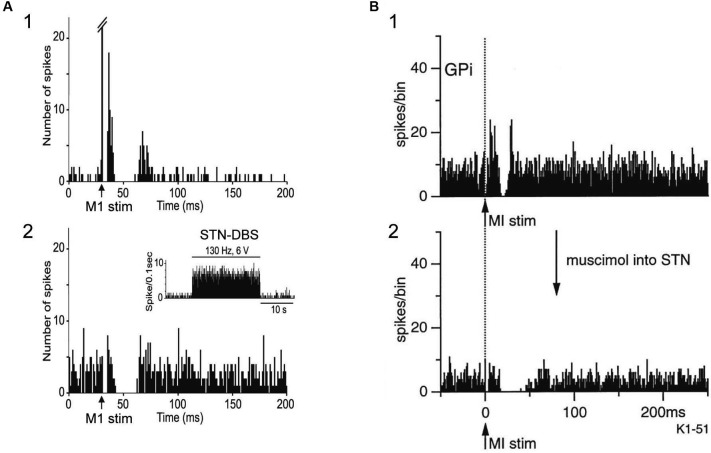
**Both STN-DBS and STN blocking disrupt information flow through the STN. (A)** Effects of local STN-DBS on cortically evoked responses of a substantia nigra pars reticulata (SNr) neuron in a normal rat. PSTH (50 trials) in response to the single pulse stimulation of the Cx (arrow) **(1)** and PSTH in response to Cx stimulation during STN-DBS **(2)** are shown. The cortically evoked early and late excitation was abolished during STN-DBS, while cortically evoked inhibition was preserved. Modified from Maurice et al. (2003). **(B)** Effects of STN blocking on cortically evoked responses of a GPi neuron in a normal monkey. PSTH (100 trials) in response to the single pulse stimulation of the Cx (arrow with dotted line) **(1)** and PSTH in response to Cx stimulation after blocking STN activity by muscimol (GABA_A_ receptor agonist) injection into the STN **(2)** are shown. The cortically evoked early and late excitation was abolished after injection of muscimol into the STN, while cortically evoked inhibition was preserved. Modified from Nambu et al. (2000). Note that the pattern of cortically evoked responses of a SNr neuron during STN-DBS is similar to that of a GPi neuron after STN blocking.

STN-DBS may also interrupt neurotransmission of abnormal signals. Maurice et al. (2003) examined the effects of STN-DBS on cortically evoked responses of SNr neurons in normal rats. Cortically evoked early and late excitation was totally abolished during high intensity STN-HFS, and much reduced during low intensity STN-DBS, while cortically evoked inhibition was preserved (Figure 4A), suggesting that information flow through the trans-STN pathway was blocked by STN-DBS without interrupting other pathways. The response patterns of SNr neurons during STN-DBS are similar to those of GPi neurons during STN blockade by muscimol in normal monkeys (Nambu et al., 2000; Figure 4B). Thus it is rational that STN-DBS has similar effect to lesion or silencing of the STN. In Parkinson’s disease, due to the loss of dopaminergic modulation, the information flow through the striato-GPi *direct* pathway is weakened, whereas the information flow through the striato-GPe *indirect* pathway is facilitated. Both STN-DBS and STN lesioning may alter the balance of inhibitory inputs through the *direct* pathway and excitatory inputs through the *hyperdirect* and* indirect* pathways to the GPi by disrupting information flow through the STN, and effectively alleviate bradykinesia seen in Parkinson’s disease. Similar idea, a functional disconnection of the stimulated elements, has also proposed by other groups (Anderson et al., 2006; Deniau et al., 2010; Moran et al., 2011).

## Conclusion

**Figure 5:**
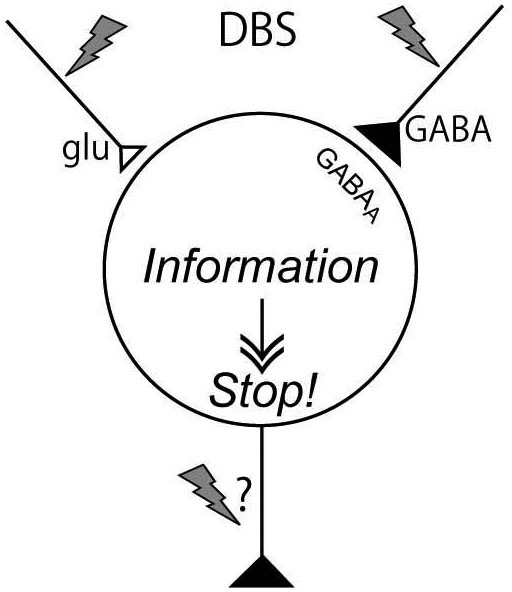
**Mechanism underling effectiveness of deep brain stimulation (DBS)**. DBS activates axon terminals in the stimulated nucleus and induces release of large amount of neurotransmitters, such as GABA and glutamate, and dissociates inputs and outputs in the stimulated nucleus, resulting in disruption of abnormal information flow through the cortico-basal ganglia loop.

DBS has variety of effects on neurons in the stimulated nucleus of the cortico-basal ganglia loop, though transmitter release, orthodromic activation of efferent axons, antidromic activation of afferent axons, and direct stimulation of passing axons nearby the stimulating electrode. The effects vary depending on the neural composition of the stimulated nucleus, and the effects extend much wider than originally expected. However, a common mechanism would underlie the effectiveness of DBS: DBS dissociates inputs and outputs in the stimulated nucleus and disrupts abnormal information flow through the cortico-basal ganglia loop (Figure 5). The mechanism may explain the paradox that DBS produces similar therapeutic effects to lesions or silencing of the nucleus.

## Conflict of interest statement

The authors declare that the research was conducted in the absence of any commercial or financial relationships that could be construed as a potential conflict of interest.

## References

[B1] Abosch A. Kapur S. Lang A. E. Hussey D. Sime E. Miyasaki J. (2003). Stimulation of the subthalamic nucleus in Parkinson’s disease does not produce striatal dopamine release. Neurosurgery 53, 1095–1105 10.1227/01.neu.0000088662.69419.1b 14580276

[B3] Anderson T. R. Hu B. Iremonger K. Kiss Z. H. (2006). Selective attenuation of afferent synaptic transmission as a mechanism of thalamic deep brain stimulation-induced tremor arrest. J. Neurosci. 26, 841–850 10.1523/jneurosci.3523-05.2006 16421304 PMC6675364

[B2] Anderson M. E. Postupna N. Ruffo M. (2003). Effects of high-frequency stimulation in the internal globus pallidus on the activity of thalamic neurons in the awake monkey. J. Neurophysiol. 89, 1150–1160 10.1152/jn.00475.2002 12574488

[B4] Aziz T. Z. Peggs D. Sambrook M. A. Crossman A. R. (1991). Lesion of the subthalamic nucleus for the alleviation of 1-methyl-4-phenyl-1,2,3,6-tetrahydropyridine (MPTP)-induced parkinsonism in the primate. Mov. Disord. 6, 288–292 10.1002/mds.870060404 1758446

[B5] Bar-Gad I. Elias S. Vaadia E. Bergman H. (2004). Complex locking rather than complete cessation of neuronal activity in the globus pallidus of a 1-methyl-4-phenyl-1,2,3,6-tetrahydropyridine-treated primate in response to pallidal microstimulation. J. Neurosci. 24, 7410–7419 10.1523/jneurosci.1691-04.2004 15317866 PMC6729780

[B6] Bekar L. Libionka W. Tian G. F. Xu Q. Torres A. Wang X. (2008). Adenosine is crucial for deep brain stimulation-mediated attenuation of tremor. Nat. Med. 14, 75–80 10.1038/nm1693 18157140

[B95] Benabid A. L. Pollak P. Gao D. Hoffmann D. Limousin P. Gay E. (1996). Chronic electrical stimulation of the ventralis intermedius nucleus of the thalamus as a treatment of movement disorders. J. Neurosurg. 84, 203–214 10.3171/jns.1996.84.2.0203 8592222

[B7] Benabid A. L. Pollak P. Gervason C. Hoffmann D. Gao D. M. Hommel M. (1991). Long-term suppression of tremor by chronic stimulation of the ventral intermediate thalamic nucleus. Lancet 337, 403–406 10.1016/0140-6736(91)91175-t 1671433

[B8] Benabid A. L. Pollak P. Gross C. Hoffmann D. Benazzouz A. Gao D. M. (1994). Acute and long-term effects of subthalamic nucleus stimulation in Parkinson’s disease. Stereotact. Funct. Neurosurg. 62, 76–84 10.1159/000098600 7631092

[B9] Benazzouz A. Gross C. Féger J. Boraud T. Bioulac B. (1993). Reversal of rigidity and improvement in motor performance by subthalamic high-frequency stimulation in MPTP-treated monkeys. Eur. J. Neurosci. 5, 382–389 10.1111/j.1460-9568.1993.tb00505.x 8261116

[B93] Bergman H. Feingold A. Nini A. Raz A. Slovin H. Abeles M. (1998). Physiological aspects of information processing in the basal ganglia of normal and parkinsonian primates. Trends Neurosci. 21, 32–38 10.1016/S0166-2236(97)01151-X 9464684

[B10] Bergman H. Wichmann T. DeLong M. R. (1990). Reversal of experimental parkinsonism by lesions of the subthalamic nucleus. Science 249, 1436–1438 10.1126/science.2402638 2402638

[B11] Beurrier C. Bioulac B. Audin J. Hammond C. (2001). High-frequency stimulation produces a transient blockade of voltage-gated currents in subthalamic neurons. J. Neurophysiol. 85, 1351–1356 11287459 10.1152/jn.2001.85.4.1351

[B12] Boertien T. Zrinzo L. Kahan J. Jahanshahi M. Hariz M. Mancini L. (2011). Functional imaging of subthalamic nucleus deep brain stimulation in Parkinson’s disease. Mov. Disord. 26, 1835–1843 10.1002/mds.23788 21674623

[B13] Boraud T. Bezard E. Bioulac B. Gross C. (1996). High frequency stimulation of the internal globus pallidus (GPi) simultaneously improves parkinsonian symptoms and reduces the firing frequency of GPi neurons in the MPTP-treated monkey. Neurosci. Lett. 215, 17–20 10.1016/s0304-3940(96)12943-8 8880743

[B14] Brotchie J. M. Mitchell I. J. Sambrook M. A. Crossman A. R. (1991). Alleviation of parkinsonism by antagonism of excitatory amino acid transmission in the medial segment of the globus pallidus in rat and primate. Mov. Disord. 6, 133–138 10.1002/mds.870060208 1647492

[B94] Brown P. (2007). Abnormal oscillatory synchronisation in the motor system leads to impaired movement. Curr. Opin. Neurobiol. 17, 656–664 10.1016/j.conb.2007.12.001 18221864

[B15] Chiken S. Nambu A. (2013). High-frequency pallidal stimulation disrupts information flow through the pallidum by GABAergic inhibition. J. Neurosci. 33, 2268–2280 10.1523/jneurosci.4144-11.2013 23392658 PMC6619164

[B16] Chiken S. Shashidharan P. Nambu A. (2008). Cortically-evoked long-lasting inhibition of pallidal neurons in a transgenic mouse model of dystonia. J. Neurosci. 28, 13967–13977 10.1523/jneurosci.3834-08.2008 19091985 PMC2702121

[B17] Coubes P. Cif L. El Fertit H. Hemm S. Vayssiere N. Serrat S. (2004). Electrical stimulation of the globus pallidus internus in patients with primary generalized dystonia: long-term results. J. Neurosurg. 101, 189–194 10.3171/jns.2004.101.2.0189 15309907

[B62] Deep-Brain Stimulation for Parkinson’s Disease Study Group (2001). Deep-brain stimulation of the subthalamic nucleus or the pars interna of the globus pallidus in Parkinson’s disease. N. Engl. J. Med. 345, 956–963 10.1056/nejmoa000827 11575287

[B18] Degos B. Deniau J. M. Chavez M. Maurice N. (2013). Subthalamic nucleus high-frequency stimulation restores altered electrophysiological properties of cortical neurons in parkinsonian rat. PLoS One 8:e83608 10.1371/journal.pone.0083608 24391793 PMC3877054

[B19] Deniau J. M. Degos B. Bosch C. Maurice N. (2010). Deep brain stimulation mechanisms: beyond the concept of local functional inhibition. Eur. J. Neurosci. 32, 1080–1091 10.1111/j.1460-9568.2010.07413.x 21039947

[B20] Dostrovsky J. O. Levy R. Wu J. P. Hutchison W. D. Tasker R. R. Lozano A. M. (2000). Microstimulation-induced inhibition of neuronal firing in human globus pallidus. J. Neurophysiol. 84, 570–574 10899228 10.1152/jn.2000.84.1.570

[B21] Dostrovsky J. O. Lozano A. M. (2002). Mechanisms of deep brain stimulation. Mov. Disord. 17, S63–S68 10.1002/mds.10143 11948756

[B22] Erez Y. Czitron H. McCairn K. Belelovsky K. Bar-Gad I. (2009). Short-term depression of synaptic transmission during stimulation in the globus pallidus of 1-methyl-4-phenyl-1,2,3,6-tetrahydropyridine-treated primates. J. Neurosci. 29, 7797–7802 10.1523/jneurosci.0401-09.2009 19535591 PMC6665635

[B23] Fellin T. Pascual O. Haydon P. G. (2006). Astrocytes coordinate synaptic networks: balanced excitation and inhibition. Physiology (Bethesda) 21, 208–215 10.1152/physiol.00161.2005 16714479

[B24] Filali M. Hutchison W. D. Palter V. N. Lozano A. M. Dostrovsky J. O. (2004). Stimulation-induced inhibition of neuronal firing in human subthalamic nucleus. Exp. Brain Res. 156, 274–281 10.1007/s00221-003-1784-y 14745464

[B25] Galati S. Mazzone P. Fedele E. Pisani A. Peppe A. Pierantozzi M. (2006). Biochemical and electrophysiological changes of substantia nigra pars reticulata driven by subthalamic stimulation in patients with Parkinson’s disease. Eur. J. Neurosci. 23, 2923–2928 10.1111/j.1460-9568.2006.04816.x 16819981

[B26] Gradinaru V. Mogri M. Thompson K. R. Henderson J. M. Deisseroth K. (2009). Optical deconstruction of parkinsonian neural circuitry. Science 324, 354–359 10.1126/science.1167093 19299587 PMC6744370

[B27] Graham W. C. Robertson R. G. Sambrook M. A. Crossman A. R. (1990). Injection of excitatory amino acid antagonists into the medial pallidal segment of a 1-methyl-4-phenyl-1,2,3,6-tetrahydropyridine (MPTP) treated primate reverses motor symptoms of parkinsonism. Life Sci. 47, PL91–PL97 10.1016/0024-3205(90)90376-3 2250573

[B28] Hammond C. Bergman H. Brown P. (2007). Pathological synchronization in Parkinson’s disease: networks, models and treatments. Trends Neurosci. 30, 357–364 10.1016/j.tins.2007.05.004 17532060

[B29] Hashimoto T. Elder C. M. Okun M. S. Patrick S. K. Vitek J. L. (2003). Stimulation of the subthalamic nucleus changes the firing pattern of pallidal neurons. J. Neurosci. 23, 1916–1923 12629196 10.1523/JNEUROSCI.23-05-01916.2003 PMC6741976

[B30] Hashimoto T. Elder C. M. Vitek J. L. (2002). A template subtraction method for stimulus artifact removal in high-frequency deep brain stimulation. J. Neurosci. Methods 113, 181–186 10.1016/s0165-0270(01)00491-5 11772439

[B31] Hershey T. Revilla F. J. Wernle A. R. McGee-Minnich L. Antenor J. V. Videen T. O. (2003). Cortical and subcortical blood flow effects of subthalamic nucleus stimulation in PD. Neurology 61, 816–821 10.1212/01.wnl.0000083991.81859.73 14504327

[B32] Hilker R. Voges J. Ghaemi M. Lehrke R. Rudolf J. Koulousakis A. (2003). Deep brain stimulation of the subthalamic nucleus does not increase the striatal dopamine concentration in parkinsonian humans. Mov. Disord. 18, 41–48 10.1002/mds.10297 12518299

[B33] Jantz J. J. Watanabe M. (2013). Pallidal deep brain stimulation modulates afferent fibers, efferent fibers and glia. J. Neurosci. 33, 9873–9875 10.1523/jneurosci.1471-13.2013 23761881 PMC6618401

[B34] Jech R. Urgosík D. Tintera J. Nebuzelský A. Krásenský J. Liscák R. (2001). Functional magnetic resonance imaging during deep brain stimulation: a pilot study in four patients with Parkinson’s disease. Mov. Disord. 16, 1126–1132 10.1002/mds.1217 11748747

[B35] Johnson M. D. McIntyre C. C. (2008). Quantifying the neural elements activated and inhibited by globus pallidus deep brain stimulation. J. Neurophysiol. 100, 2549–2563 10.1152/jn.90372.2008 18768645 PMC2585404

[B36] Johnson M. D. Miocinovic S. McIntyre C. C. Vitek J. L. (2008). Mechanisms and targets of deep brain stimulation in movement disorders. Neurotherapeutics 5, 294–308 10.1016/j.nurt.2008.01.010 18394571 PMC2517242

[B37] Kita H. Kita T. (2011). Cortical stimulation evokes abnormal responses in the dopamine-depleted rat basal ganglia. J. Neurosci. 31, 10311–10322 10.1523/jneurosci.0915-11.2011 21753008 PMC3138188

[B38] Koller W. Pahwa R. Busenbark K. Hubble J. Wilkinson S. Lang A. (1997). High-frequency unilateral thalamic stimulation in the treatment of essential and parkinsonian tremor. Ann. Neurol. 42, 292–299 10.1002/ana.410420304 9307249

[B39] Kravitz A. V. Freeze B. S. Parker P. R. L. Kay K. Thwin M. T. Deisseroth K. (2010). Regulation of parkinsonian motor behaviours by optogenetic control of basal ganglia circuitry. Nature 466, 622–626 10.1038/nature09159 20613723 PMC3552484

[B40] Kringelbach M. L. Jenkinson N. Owen S. L. Aziz T. Z. (2007). Translational principles of deep brain stimulation. Nat. Rev. Neurosci. 8, 623–635 10.1038/nrn2196 17637800

[B41] Lafreniere-Roula M. Kim E. Hutchison W. D. Lozano A. M. Hodaie M. Dostrovsky J. O. (2010). High-frequency microstimulation in human globus pallidus and substantia nigra. Exp. Brain Res. 205, 251–261 10.1007/s00221-010-2362-8 20640411

[B42] Leblois A. Reese R. Labarre D. Hamann M. Richter A. Boraud T. (2010). Deep brain stimulation changes basal ganglia output nuclei firing pattern in the dystonic hamster. Neurobiol. Dis. 38, 288–298 10.1016/j.nbd.2010.01.020 20138992

[B43] Lee K. H. Chang S. Y. Roberts D. W. Kim U. (2004). Neurotransmitter release from high-frequency stimulation of the subthalamic nucleus. J. Neurosurg. 101, 511–517 10.3171/jns.2004.101.3.0511 15352610

[B44] Levy R. Lang A. E. Dostrovsky J. O. Pahapill P. Romas J. Saint-Cyr J. (2001). Lidocaine and muscimol microinjections in subthalamic nucleus reverse Parkinsonian symptoms. Brain 124, 2105–2118 10.1093/brain/124.10.2105 11571226

[B45] Li S. Arbuthnott G. W. Jutras M. J. Goldberg J. A. Jaeger D. (2007). Resonant antidromic cortical circuit activation as a consequence of high-frequency subthalamic deep-brain stimulation. J. Neurophysiol. 98, 3525–3537 10.1152/jn.00808.2007 17928554

[B46] Li Q. Ke Y. Chan D. C. Qian Z. M. Yung K. K. Ko H. (2012). Therapeutic deep brain stimulation in Parkinsonian rats directly influences motor cortex. Neuron 76, 1030–1041 10.1016/j.neuron.2012.09.032 23217750

[B47] Limousin P. Pollak P. Benazzouz A. Hoffmann D. Le Bas J. F. Broussolle E. (1995). Effect of parkinsonian signs and symptoms of bilateral subthalamic nucleus stimulation. Lancet 345, 91–95 10.1016/s0140-6736(95)90062-4 7815888

[B48] Liu Y. Postupna N. Falkenberg J. Anderson M. E. (2008). High frequency deep brain stimulation: what are the therapeutic mechanisms? Neurosci. Biobehav. Rev. 32, 343–351 10.1016/j.neubiorev.2006.10.007 17187859

[B49] Maurice N. Thierry A. M. Glowinski J. Deniau J. M. (2003). Spontaneous and evoked activity of substantia nigra pars reticulata neurons during high-frequency stimulation of the subthalamic nucleus. J. Neurosci. 23, 9929–9936 14586023 10.1523/JNEUROSCI.23-30-09929.2003 PMC6740874

[B50] McCairn K. W. Turner R. S. (2009). Deep brain stimulation of the globus pallidus internus in the parkinsonian primate: local entrainment and suppression of low-frequency oscillations. J. Neurophysiol. 101, 1941–1960 10.1152/jn.91092.2008 19164104 PMC3350155

[B51] McIntyre C. C. Savasta M. Walter B. L. Vitek J. L. (2004). How does deep brain stimulation work? Present understanding and future questions. J. Clin. Neurophysiol. 21, 40–50 10.1097/00004691-200401000-00006 15097293

[B52] Meissner W. Harnack D. Reese R. Paul G. Reum T. Ansorge M. (2003). High-frequency stimulation of the subthalamic nucleus enhances striatal dopamine release and metabolism in rats. J. Neurochem. 85, 601–609 10.1046/j.1471-4159.2003.01665.x 12694386

[B53] Meissner W. Leblois A. Hansel D. Bioulac B. Gross C. E. Benazzouz A. (2005). Subthalamic high frequency stimulation resets subthalamic firing and reduces abnormal oscillations. Brain 128, 2372–2382 10.1093/brain/awh616 16123144

[B54] Mink J. W. (1996). The basal ganglia: focused selection and inhibition of competing motor programs. Prog. Neurobiol. 50, 381–425 10.1016/s0301-0082(96)00042-1 9004351

[B55] Miocinovic S. Parent M. Butson C. R. Hahn P. J. Russo G. S. Vitek J. L. (2006). Computational analysis of subthalamic nucleus and lenticular fasciculus activation during therapeutic deep brain stimulation. J. Neurophysiol. 96, 1569–1580 10.1152/jn.00305.2006 16738214

[B56] Montgomery E. B. Jr. (2006). Effects of GPi stimulation on human thalamic neuronal activity. Clin. Neurophysiol. 117, 2691–2702 10.1016/j.clinph.2006.08.011 17029953

[B57] Moran A. Stein E. Tischler H. Belelovsky K. Bar-Gad I. (2011). Dynamic stereotypic responses of Basal Ganglia neurons to subthalamic nucleus high-frequency stimulation in the parkinsonian primate. Front. Syst. Neurosci. 5:21 10.3389/fnsys.2011.00021 21559345 PMC3085177

[B58] Nambu A. (2007). Globus pallidus internal segment. Prog. Brain. Res. 160, 135–150 10.1016/s0079-6123(06)60008-3 17499112

[B59] Nambu A. Tokuno H. Takada M. (2002). Functional significance of the cortico-subthalamo-pallidal ‘hyperdirect’ pathway. Neurosci. Res. 43, 111–117 10.1016/s0168-0102(02)00027-5 12067746

[B60] Nambu A. Tokuno H. Hamada I. Kita H. Imanishi M. Akazawa T. (2000). Excitatory cortical inputs to pallidal neurons via the subthalamic nucleus in the monkey. J. Neurophysiol. 84, 289–300 10899204 10.1152/jn.2000.84.1.289

[B61] Nishibayashi H. Ogura M. Kakishita K. Tanaka S. Tachibana Y. Nambu A. (2011). Cortically evoked responses of human pallidal neurons recorded during stereotactic neurosurgery. Mov. Disord. 26, 469–476 10.1002/mds.23502 21312279

[B63] Ostrem J. L. Starr P. A. (2008). Treatment of dystonia with deep brain stimulation. Neurotherapeutics 5, 320–330 10.1016/j.nurt.2008.01.002 18394573 PMC5084173

[B64] Pralong E. Debatisse D. Maeder M. Vingerhoets F. Ghika J. Villemure J. G. (2003). Effect of deep brain stimulation of GPI on neuronal activity of the thalamic nucleus ventralis oralis in a dystonic patient. Neurophysiol. Clin. 33, 169–173 10.1016/j.neucli.2003.07.001 14519544

[B65] Ranck J. B. Jr. (1975). Which elements are excited in electrical stimulation of mammalian central nervous system: a review. Brain Res. 98, 417–440 10.1016/0006-8993(75)90364-9 1102064

[B66] Reese R. Leblois A. Steigerwald F. Potter-Nerger M. Herzog J. Mehdorn H. M. (2011). Subthalamic deep brain stimulation increases pallidal firing rate and regularity. Exp. Neurol. 229, 517–521 10.1016/j.expneurol.2011.01.020 21303674

[B67] Rehncrona S. Johnels B. Widner H. Törnqvist A. L. Hariz M. Sydow O. (2003). Long-term efficacy of thalamic deep brain stimulation for tremor: double-blind assessments. Mov. Disord. 18, 163–170 10.1002/mds.10309 12539209

[B68] Sano H. Chiken S. Hikida T. Kobayashi K. Nambu A. (2013). Signals through the striatopallidal indirect pathway stop movements by phasic excitation in the substantia nigra. J. Neurosci. 33, 7583–7594 10.1523/jneurosci.4932-12.2013 23616563 PMC6619573

[B69] Shi L. H. Luo F. Woodward D. J. Chang J. Y. (2006). Basal ganglia neural responses during behaviorally effective deep brain stimulation of the subthalamic nucleus in rats performing a treadmill locomotion test. Synapse 59, 445–457 10.1002/syn.20261 16521122

[B70] Shin D. S. Samoilova M. Cotic M. Zhang L. Brotchie J. M. Carlen P. L. (2007). High frequency stimulation or elevated K+ depresses neuronal activity in the rat entopeduncular nucleus. Neuroscience 149, 68–86 10.1016/j.neuroscience.2007.06.055 17826920

[B71] Shink E. Smith Y. (1995). Differential synaptic innervation of neurons in the internal and external segments of the globus pallidus by the GABA- and glutamate-containing terminals in the squirrel monkey. J. Comp. Neurol. 358, 119–141 10.1002/cne.903580108 7560274

[B72] Shon Y. M. Lee K. H. Goerss S. J. Kim I. Y. Kimble C. Van Gompel J. J. (2010). High frequency stimulation of the subthalamic nucleus evokes striatal dopamine release in a large animal model of human DBS neurosurgery. Neurosci. Lett. 475, 136–140 10.1016/j.neulet.2010.03.060 20347936 PMC2874873

[B73] Siegfried J. Lippitz B. (1994a). Bilateral chronic electrostimulation of ventroposterolateral pallidum: a new therapeutic approach for alleviating all parkinsonian symptoms. Neurosurgery 35, 1126–1130 10.1227/00006123-199412000-00016 7885558

[B74] Siegfried J. Lippitz B. (1994b). Chronic electrical stimulation of the VL-VPL complex and of the pallidum in the treatment of movement disorders: personal experience since 1982. Stereotact. Funct. Neurosurg. 62, 71–75 10.1159/000098599 7631091

[B75] Smith Y. Wichmann T. DeLong M. R. (1994). Synaptic innervation of neurones in the internal pallidal segment by the subthalamic nucleus and the external pallidum in monkeys. J. Comp. Neurol. 343, 297–318 10.1002/cne.903430209 8027445

[B76] Starr P. A. Rau G. M. Davis V. Marks W. J. Jr. Ostrem J. L. Simmons D. (2005). Spontaneous pallidal neuronal activity in human dystonia: comparison with Parkinson’s disease and normal macaque. J. Neurophysiol. 93, 3165–3176 10.1152/jn.00971.2004 15703229

[B77] Stefani A. Fedele E. Galati S. Pepicelli O. Frasca S. Pierantozzi M. (2005). Subthalamic stimulation activates internal pallidus: evidence from cGMP microdialysis in PD patients. Ann. Neurol. 57, 448–452 10.1002/ana.20402 15732123

[B78] Tachibana Y. Iwamuro H. Kita H. Takada M. Nambu A. (2011). Subthalamo-pallidal interactions underlying parkinsonian neuronal oscillations in the primate basal ganglia. Eur. J. Neurosci. 34, 1470–1484 10.1111/j.1460-9568.2011.07865.x 22034978

[B79] Tai C. H. Boraud T. Bezard E. Bioulac B. Gross C. Benazzouz A. (2003). Electrophysiological and metabolic evidence that high-frequency stimulation of the subthalamic nucleus bridles neuronal activity in the subthalamic nucleus and the substantia nigra reticulata. FASEB J. 17, 1820–1830 10.1096/fj.03-0163com 14519661

[B80] Tawfik V. L. Chang S. Y. Hitti F. L. Roberts D. W. Leiter J. C. Jovanovic S. (2010). Deep brain stimulation results in local glutamate and adenosine release: investigation into the role of astrocytes. Neurosurgery 67, 367–375 10.1227/01.neu.0000371988.73620.4c 20644423 PMC2919357

[B81] Vedam-Mai V. van Battum E. Y. Kamphuis W. Feenstra M. G. Denys D. Reynolds B. A. (2012). Deep brain stimulation and the role of astrocytes. Mol. Psychiatry 17, 124–131 10.1038/mp.2011.61 21625231

[B82] Vidailhet M. Jutras M. F. Roze E. Grabli D. (2013). Deep brain stimulation for dystonia. Handb. Clin. Neurol. 116, 167–187 10.1016/B978-0-444-53497-2.00014-0 24112893

[B83] Vitek J. L. (2008). Deep brain stimulation: how does it work? Cleve. Clin. J. Med. 75(Suppl. 2), S59–S65 10.3949/ccjm.75.suppl_2.s59 18540149

[B84] Wang L. Y. Kaczmarek L. K. (1998). High-frequency firing helps replenish the readily releasable pool of synaptic vesicles. Nature 394, 384–388 10.1038/28645 9690475

[B85] Welter M. L. Houeto J. L. Bonnet A. M. Bejjani P. B. Mesnage V. Dormont D. (2004). Effects of high-frequency stimulation on subthalamic neuronal activity in parkinsonian patients. Arch. Neurol. 61, 89–96 10.1001/archneur.61.1.89 14732625

[B86] Wichmann T. (2000). A digital averaging method for removal of stimulus artifacts in neurophysiologic experiments. J. Neurosci. Methods 98, 57–62 10.1016/s0165-0270(00)00190-4 10837871

[B88] Wichmann T. Bergman H. DeLong M. R. (1994). The primate subthalamic nucleus. III. Changes in motor behavior and neuronal activity in the internal pallidum induced by subthalamic inactivation in the MPTP model of parkinsonism. J. Neurophysiol. 72, 521–530 7983516 10.1152/jn.1994.72.2.521

[B87] Wichmann T. Delong M. R. (2006). Deep brain stimulation for neurologic and neuropsychiatric disorders. Neuron 52, 197–204 10.1016/j.neuron.2006.09.022 17015236

[B89] Windels F. Bruet N. Poupard A. Urbain N. Chouvet G. Feuerstein C. (2000). Effects of high frequency stimulation of subthalamic nucleus on extracellular glutamate and GABA in substantia nigra and globus pallidus in the normal rat. Eur. J. Neurosci. 12, 4141–4146 10.1046/j.1460-9568.2000.00296.x 11069610

[B90] Windels F. Carcenac C. Poupard A. Savasta M. (2005). Pallidal origin of GABA release within the substantia nigra pars reticulata during high-frequency stimulation of the subthalamic nucleus. J. Neurosci. 25, 5079–5086 10.1523/jneurosci.0360-05.2005 15901790 PMC6724863

[B91] Wu Y. R. Levy R. Ashby P. Tasker R. R. Dostrovsky J. O. (2001). Does stimulation of the GPi control dyskinesia by activating inhibitory axons? Mov. Disord. 16, 208–216 10.1002/mds.1046 11295772

[B92] Zucker R. S. Regehr W. G. (2002). Short-term synaptic plasticity. Annu. Rev. Physiol. 64, 355–405 10.1146/annurev.physiol.64.092501.114547 11826273

